# Enzymatic Processes Triggered by PEF for Astaxanthin Extraction From *Xanthophyllomyces dendrorhous*

**DOI:** 10.3389/fbioe.2020.00857

**Published:** 2020-07-29

**Authors:** Diederich Aguilar-Machado, Carlota Delso, Juan Manuel Martinez, Lourdes Morales-Oyervides, Julio Montañez, Javier Raso

**Affiliations:** ^1^Food Technology, Facultad de Veterinaria, Universidad de Zaragoza, Zaragoza, Spain; ^2^Department of Food Research, Universidad Autónoma de Coahuila, Saltillo, Mexico; ^3^Department of Chemical Engineering, Universidad Autónoma de Coahuila, Saltillo, Mexico

**Keywords:** astaxanthin, pulsed electric field, extraction, *Xanthophyllomyces dendrorhous*, esterase, enzymatic activity

## Abstract

The aim of this study was to evaluate the potential of pulsed electric fields (PEF) to improve the extraction of the lipid-soluble astaxanthin from fresh biomass of a wild-type (CECT 11028) and mutant (ATCC 74219) *Xanthophyllomyces dendrorhous* strain using ethanol as solvent. Inactivation and propidium uptake studies revealed that inactivation is a good index for estimated the proportion of irreversible permeabilized cells when inactivation is higher than 70% in the two strains. Ethanol was ineffective for extracting carotenoids from the PEF-treated cells (20 kV/cm, 135 μs) of the two strains. However, after aqueous incubation of PEF-treated *X. dendrorhous* ATCC 74219 cells for 12 h, up to 2.4 ± 0.05 mg/g dried weight (d.w.) of carotenoids were extracted in ethanol. From total carotenoid extracted, around 84% corresponded to all-trans astaxanthin. The detection and quantification of esterase activity in the supernatant and the relationship between the percentage of esterase activity quantified and the amount of carotenoids extracted indicate that the extraction of astaxanthin was mediated by enzymatic esterase activity triggered by PEF during incubation. On the other hand, the formation of a large lipid globule into the cytoplasm of PEF-treated *X. dendrorhous* CECT 11028 cells during aqueous incubation prevented carotenoid extraction. The process developed in this investigation represents a more sustainable and greener method that those previously used for extracting astaxanthin from yeast.

## Introduction

Astaxanthin (3,3′-dihydroxy-β, β-carotene-4,4′-dione; C_40_H_5_2O_4_) is a lipid-soluble oxycarotenoid widely used in food, aquaculture, nutraceutical, cosmetic and pharmaceutical industries due to its relatively high antioxidant activity compared with other antioxidant molecules. It has been reported that astaxanthin has 10 times the antioxidant activity of β-carotene, 60 times of coenzyme Q10, and 1000 times more antioxidant activity than vitamin E (Higuera-Ciapara et al., 2006; Shah et al., 2016; Zhao et al., 2019). Astaxanthin unique chemical structure makes it a powerful antioxidant with extraordinary biological and physiological properties including cardiovascular disease prevention, strengthening the immune system and anti-tumoral, anti-inflammatory, and anti-diabetes effects (Kurihara et al., 2002; Uchiyama et al., 2002; Ohgami et al., 2003; Pashkow et al., 2008; Nagendraprabhu and Sudhandiran, 2011; Peng et al., 2012; Fakhri et al., 2018).

Currently, the main use of astaxanthin is as a feed additive for providing the characteristic pink/red color of salmons, trout, and crustaceans. Around 99% of astaxanthin available in the market is produced synthetically due to their low production cost, high purity, and high stability, and only around 1% of astaxanthin derived from a natural source. The total astaxanthin production was about 200 tons in 2014 worth 368 million euros and this is expected to encrease double in 2022 (Molino et al., 2018). However, synthetic astaxanthin has not been approved for human consumption by safety concerns related to the use of petrochemicals during its synthesis process (Li et al., 2011; Shah et al., 2016). On the other hand, natural astaxanthin has gained huge attention from researches and consumers in the last years. Studies have suggested that natural astaxanthin has around 20 times more antioxidant activity compared with the synthetic molecule (Capelli et al., 2013). The growing demand from industries and consumers has encouraged researches to demonstrate the feasibility of astaxanthin production and extraction from diverse natural sources.

The yeast *Xanthophyllomyces dendrorhous* is considered one of the most important sources of natural astaxanthin because around 84% of the total carotenoids that it synthesizes correspond to these compound (Stoklosa et al., 2018). Astaxanthin is formed as different geometrical isomers with cis and trans double bonds in the polyene chain. The yeast *X. dendrorhous* synthesize the all-trans isomer mainly, but the 9-cis and 13-cis isomers can be also found (Schmidt et al., 2011). The high-rate cell growth with short cultivation cycles, the ability to metabolize waste-based culture media, no seasonal conditions constraints and the easy manipulation of culture conditions to improve yields make the yeast *X. dendrorhous* an alternative for large scale production of natural astaxanthin (Wu et al., 2018; Villegas-Méndez et al., 2019). Generally astaxanthin production from naturally isolated strains of *X. dendrorhous* is very low (between 200 and 400 μg/g), however, some authors have developed genetic manipulations to get hyper-producer strains with yields up to 10-fold higher than the wild-type strains (Visser et al., 2003; Zhuang et al., 2020).

*Xanthophyllomyces dendrorhous* accumulates astaxanthin in the cytoplasm in the form of droplets associated with the cytoplasmatic membrane and fatty acids (Johnson and Gil-Hwan, 1991; Jacobson et al., 1995; Urnau et al., 2018). The presence of a thick, rigid and indigestible cell envelope, as well as the lipophilic nature of astaxanthin, are the biggest challenges in the development of efficient methodologies for extraction of this compound (Gogate and Nadar, 2015). Over the last years, several cell wall disruption processes such as chemical (acid extraction), enzymatic and physical or mechanical (ultrasound, microwave, high-pressure homogenization) methods have been evaluated to facilitate the recovery of intracellular carotenoids from *X. dendrorhous* (Choi et al., 2007; Ni et al., 2008; Michelon et al., 2012; Gogate and Nadar, 2015; Hasan et al., 2016; Urnau et al., 2018; Zhuang et al., 2020). However, those techniques present some disadvantages such as high implementation costs, long extraction time, excessive cell destruction and high cost of purification of the target molecule (Martínez et al., 2018). Furthermore, most carotenoids extraction techniques require the drying of biomass with subsequent extraction with a large amount of organic solvent. Therefore, it is desirable to develop efficient extraction methods from wet biomass using green solvents in order to achieve economical and eco-friendly processes (Martínez et al., 2019).

Pulsed electric fields (PEF) is a technique that consists in the application of an external electric field to induce the permeabilization (electroporation) of biological membranes (Kotnik et al., 2015). In contrast to other technologies that cause cell disruption, energetic requirements of PEF are low, it is easily scalable, does not result in cell disintegration, minimizing the release of cell debris and facilitating purification of the extracted compound (Kotnik et al., 2015).

Several studies have demonstrated that electroporation by PEF improve the extraction of intracellular components such as proteins, lipids, and pigments from microorganisms has been reported (Ganeva et al., 2003; Chittapun et al., 2020; Leonhardt et al., 2020). Very recently, it has been proposed that PEF not only permeabilized the cytoplasmatic membrane to enable the release of intracellular biomolecules but also trigger some enzymatic process that accelerates the autolysis and the subsequent release of intracellular components (Martínez et al., 2018, 2019; Silve et al., 2018; Scherer et al., 2019). The application of PEF technology on the recovery of intra-cellular carotenoids from *X. dendrorhous* has not been reported.

This study aimed to evaluate the effect of PEF-assisted on the extraction of astaxanthin from fresh biomass of a wild-type and a mutant hyper-producer *X. dendrorhous* strain using ethanol as solvent.

## Materials and Methods

### Yeast Strains and Culture Conditions

The wild-type *X. dendrorhous* CECT 11028 and the mutant hyper-producer *X. dendrorhous* ATCC 74219 strains were obtained from the Colección Española de Cultivos Tipo (CECT) and the American Type Culture Collection (ATCC, Beltsville, MD, United States), respectively. The microorganisms were maintained in cryovials at –80°C. Yeasts were grown in 500 mL glass flask containing 250 mL of Potato-Dextrose Broth (PDB, Oxoid, Basingstoke, United Kingdom) for 6 days in a rotatory shaker (Heidolph Unimax 1010, Germany) at 200 rpm and 25°C. The inoculum was around 10^6^ cells/mL defined by Thoma counting chamber.

Yeast growth was monitored by measuring the cell density at 600 nm and the cell number by the plate-counting method (PDA, Oxoid, Basingstoke, United Kingdom). Incubation time needed to obtain the maximum carotenoid production was established by determining the carotenoid production during the cultivation period using the dimethyl sulfoxide method as described below.

### PEF Treatments

Pulsed electric fields unit (Modulator PG, ScandiNova, Uppsala, Sweden) used in this investigation was previously described by Saldaña et al. (2010). Before the treatments, fresh biomass of *X. dendrorhous* CECT 11028 and *X. dendrorhous* ATCC 74219 were centrifuged (Heraeus Megafuge 1.0R, United Kingdom) at 3000 × *g* for 5 min at 4°C. Next, the pellet was resuspended in a citrate-phosphate McIlvaine buffer (pH 7.0, 1 mS/cm) to a final concentration of approximately 10^8^ cell/mL. The cell suspension (0.5 mL) of each strain was deposited in a static parallel-electrode tempered chamber (25 ± 1°C) with a gap of 0.25 cm and a radius of 0.8 cm employing a 1 mL sterile syringe (TERUMO, Leuven, Belgium). The cell suspension was subjected to PEF treatment of 10–60 monopolar square pulses of 3 μs pulses at electric field strength between 10 and 25 kV/cm at 0.5 Hz.

#### PEF Treatment Intensity Effect on Cell Inactivation and Irreversible Permeabilization

PEF treatment intensity was evaluated between 10 to 25 kV/cm at 0.5 Hz frequency. The specific energy applied during treatments ranged from 3.0 to 114.3 kJ/kg, corresponding to the minimum and maximum treatment intensity applied, respectively.

After PEF treatment, cells were plated in PDA and incubated at 25°C for 72 h. The inactivation degree was expressed as the logarithmic ratio of the initial number (N_o_) of cells and the number of survivors (N_t_) after different PEF treatments.

The irreversible permeabilization after PEF treatments was quantified by propidium iodide uptake technique (García-Gonzalo and Pagán, 2017). PEF-treated and untreated cells were stained after 1 h of the PEF treatments. 50 μL of PI (0.1 mg/mL) was added to 450 μL of yeast cell suspension and incubated under dark conditions for 5 min. Then, the cells were centrifugated and washed with 450 μL of phosphate-buffered saline (PBS) solution of pH 7.4 to remove the extracellular PI remained. This procedure was repeated three times.

#### PEF Treatment Intensity Effect on Carotenoid Extraction

Non-treated or PEF-treated yeast suspensions (10^8^ cell/mL) either immediately after PEF treatment or after an incubation process ranging from 3 to 24 h at 25°C in a buffer solution of pH 7.0 were centrifuged at 3,000 × *g* for 5 min and re-suspended in ethanol at 96% for 12 h. After incubation, total carotenoids were quantified following the methodology described below.

After selecting the required incubation time in the buffer solution of pH 7.0, the kinetics of carotenoids extraction were evaluated for non-treated and PEF-treated cells at different electric field strengths (10, 15, 20, and 25 kV/cm for 135 μs).

### Evaluation of the Enzymatic Activity in the Supernatant Containing Yeast

Enzymatic activity was evaluated in the supernatant of PEF-treated cells during the incubation in buffer solution. Untreated cells were used as control. Then, enzymatic thermal inactivation and its effect on carotenoids extraction were also assessed.

#### β-Glucanase Activity Measurement

β-glucanase activity (EC 3.2.1.6) in the supernatant of PEF-treated and untreated cells was conducted using the Megazyme Azo-barley β-glucan method (malt and bacterial β-glucanase and cellulase assay procedure, Megazyme, Ireland). Aliquots of 0.05 mL of Azo-Barley glucan substrate solution (pre-heated at 30°C) were dispensed into centrifuge tubes and incubated at 30°C for 5 min. After that, 1 mL of the supernatant of each yeast suspension was mixed with the pre-heated glucan substrate and incubated at 30°C for 10 min. After incubation, 3 mL of the precipitant solution [30.0 g of C_2_H_3_NaO_2_ and 3.0 g of ZnC_4_H_6_O_4_ in 1 L of ethanol (66.5%), methanol (3.5%) and water (30%)] were added and stirred vigorously. Tubes were stored at room temperature for 5 min, stirred and centrifuged (1000 × *g*, 10 min). Finally, absorbance at 590 nm of the supernatant of each sample was read against distilled water. With each set of determinations, a reaction blank was included. Enzymatic activity was calculated by correlating the malt β-glucanase standard curve on Azo Barley Glucan and absorbance from each sample.

#### Esterase Activity Measurement

The measurement of esterase enzymatic activity (EC 3.1.1.1) of supernatant of PEF-treated and untreated cells was performed with the colorimetric *p*-nitrophenyl chromogenic assay (Gilham and Lehner, 2005). For analysis, a stock solution of 250 mM of *p*-nitrophenyl-acetate dissolved in dichloromethane (Cl_2_CH_2_) was prepared. Immediately before determination, 20 μL of stock solution was diluted in 10 mL of a McIlwaine buffer (pH 8.0 and conductivity of 1 mS/cm). Then, 100 μL of the supernatant of each incubation time was added and vortexed with 1 mL of *p*-nitrophenyl-acetate solution in glass tubes and incubated at 37°C for 30 min. The liberation of p-nitrophenol was proportional to esterase activity measured spectrophotometrically at 410 nm. Distilled water was used as a blank.

#### Esterase Thermal Inactivation and Release of Carotenoids From Yeast

A suspension of PEF-treated cells (20 kV/cm and 135 μs) was heat-treated at 50 and 60°C ± 1°C for 5 min and incubated in a buffer solution of pH 7.0 for 12 h at 25°C. Preliminary experiments were conducted to define the temperatures levels required to inactivate esterase without affecting astaxanthin. After incubation, unheated and heat-treated PEF-treated cells were centrifugated and suspended in ethanol for carotenoids extraction during 24 h. The supernatant was analyzed to determine the amount of the esterase activity remaining after incubation. The percentage of esterase activity remained in the heat-treated samples was calculated according to the maximum esterase activity quantified in the unheated PEF-treated cell suspension after 12 h of incubation.

### Analytical Methods

Total carotenoids were recovered and quantified following the methodology described by Sedmak et al. (1990). 0.5 mL of Dimethyl sulfoxide (DMSO) was added to 1.0 mL of biomass previously centrifugated. Then 0.2 mL of 0.01 M sodium phosphate and 2.0 mL of hexane: ethyl acetate (1:1) was added and vortexed for 5 min. The samples were then centrifugated for 5 min at 10,000 × *g* to separate the organic phase. Carotenoids concentration was quantified spectrophotometrically for DMSO extraction and PEF assisted extraction. The total carotenoid yield was calculated using the equation:

(1) Yx=V*⁢A*⁢106E1%100*M*

where Yx represents the carotenoid yield (mg/g d.w.); A is the absorbance at 480 nm; V the volume of solvent used (mL); M the dry cell mass (g) and E^1%^ the specific absorptivity of solvent (2100).

For HPLC analysis, the ethanolic extract of each sample was previously evaporated in a vacuum concentrator and then resuspended in methanol/dichloromethane (3:1, v/v) for subsequent injection. Astaxanthin quantification was performed according to the method described by Yuan and Chen (1997). HPLC analysis was performed on a Varian ProStar high-performance liquid chromatograph equipped with a ProStar 240 ternary pump, an automatic ProStar 410 autosampler and a ProStar 335 photodiode array detector. The separation was performed using a reverse-phase column (LC Luna^®^ 100 Å C18 250 × 4.6 mm; 5 μm particle size, Phenomenex, United States) with a pre-column (LC Luna 50 × 4.6 mm; 5 μm particle size, Phenomenex, United States). A single mobile phase of methanol/dichloromethane/water (80.5:17:2.5, by volume) was used during analysis. The flow rate was 1.0 mL/min with a pre-conditioned sample at 25°C. *Trans-*astaxanthin concentration was determinate by comparing the time retention of a commercial standard of *trans-*astaxanthin (Sigma, St Louis, MO, United States) with each sample analyzed.

### Statistical Analysis

All experiments were performed in triplicate and presented as the average value ± standard deviation (SD). The differences were considered significant to *p* < 0.05. One-way analysis of variance (ANOVA) and Tukey test were used to determine the significant differences between treatment using Statistica 7.0 (Statsoft, Tulsa, OK, United States).

## Results

### Sensitivity of *X. dendrorhous* CECT 11028 and *X. dendrorhous* ATCC^®^ 74219^TM^ to Pulsed Electric Field Treatments

**FIGURE 1:**
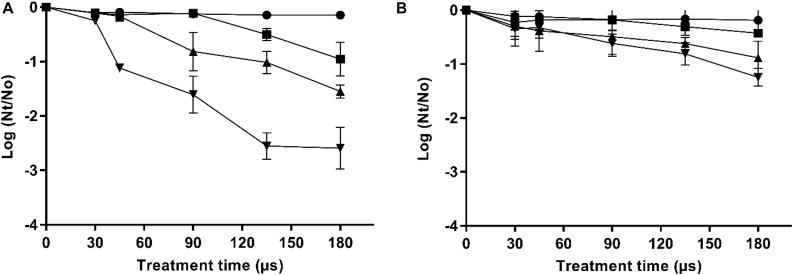
Inactivation curves of *X. dendrorhous* CECT 11028 **(A)** and *X. dendrorhous* ATCC 74219 **(B)** by PEF at different electric fields strength. 10 kV/cm (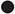), 15 kV/cm (■), 20 kV/cm (▲), 25 kV/cm (▼).

The inactivation by PEF treatments of the two *X. dendrorhous* strains used in this study (CECT 11028 and ATCC 74219) varying electric field strength and treatment time is shown in Figures 1A,B, respectively. It can be seen that the inactivation increased with the electric field strength and treatment time above the electric field threshold that was 10 and 15 kV/cm for the strains CECT 11028 and ATCC 74219, respectively. *X. dendrorhous* ATCC 74219 presented higher resistance to PEF inactivation compared to *X. dendrorhous* CECT 11028 under the most intense treatment mainly. For example, while 1.6, 2.55 and 2.56 Log_10_ cycles of inactivation were observed after 15, 20 and 25 kV/cm and 180 μs for the CECT 11028 strain, an inactivation of 0.61, 0.81 and 1.25 Log10 cycles was obtained for *X. dendrorhous* ATCC 74219.

**FIGURE 2:**
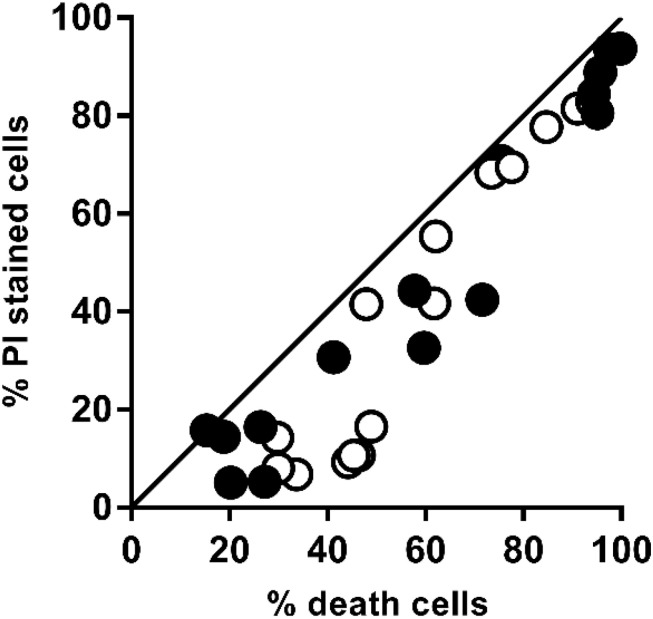
Relationship between the percentage of irreversible permeabilized cells and the percentage of dead cells of *X. dendrorhous* CECT 11028 (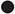) and *X. dendrorhous* ATCC 74219 (○) after applied PEF treatments shown in Figure 1. The straight line with slope 1 and intercept 0 represents a perfect agreement between the percentage of PI uptake and cell death.

**FIGURE A1:**
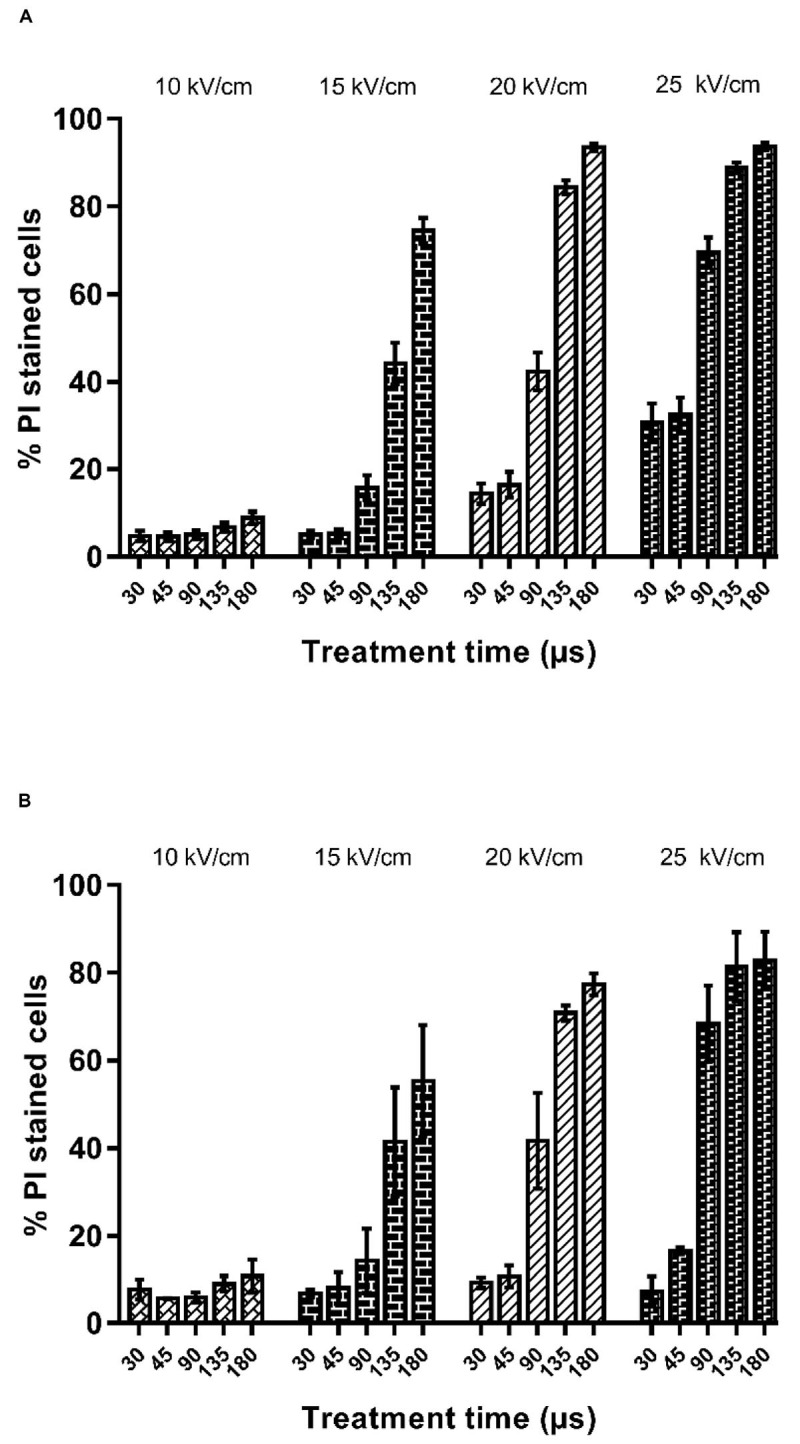
Percentage of propidium iodide uptake by *X. dendrorhous* CECT 11028 **(A)** and *X. dendrorhous* ATCC 74219 **(B)** after PEF treatments with different electric field strength and treatment time.

It is well-known that one of the effects of PEF is the loss of the selective permeability of the cell membranes as a consequence of the electroporation. Irreversible electroporation allows the uncontrolled pass of ions and macromolecules through the cell membranes leading to microbial inactivation by loss of the cellular homeostasis. Figure 2 shows the relationship between the percentage of inactivated and irreversible permeabilized cells after the application of PEF at different intensities (10–25 kV/cm) for both strains (PI results can be viewed in the Appendix Figure A1). For both microorganisms, a good correlation between irreversible electroporation and inactivation was observed when the percentage of cell death was higher than 70%. On the other hand, a higher percentage of inactivated cells compared to PI permeabilized cells were observed when the inactivation was below this value. The lack of correlation between the percentage of dead and permeabilized cells when the inactivation effect was low could indicate that a certain number of inactivated cells were able to recover the integrity of their membrane even after losing their viability (García et al., 2007; Luengo et al., 2014; Martínez et al., 2018). Therefore, this proportion of inactivated cells would behave just like those non-electroporated cells during the extraction of intracellular compounds.

### Carotenoid Extraction of *Xanthophyllomyces* Strains Assisted by PEF

According to the data obtained from the inactivation and permeabilization, a PEF treatment at 20 kV/cm for 135 μs that corresponds to a total specific energy of 54 kJ/kg was then selected to evaluate the effect of PEF in the carotenoids extraction from the two *X dendrorhous* strains. This PEF treatment resulted in an inactivation/permeabilization of around 80% in the cell population of the two strains.

Preliminary studies showed that when both untreated and PEF-treated cells were immediately suspended in ethanol, the presence of carotenoids in the extraction medium was deficient even after 48 h of incubation.

**FIGURE 3:**
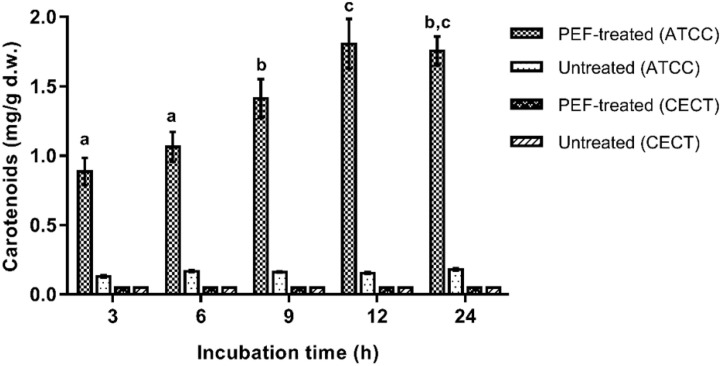
Influence of the incubation time in buffer of pH 7.0 on the extracted carotenoids in ethanol for 12 h from untreated and PEF treated cells of *X. dendrorhous* CECT 11028 and *X. dendrorhous* ATCC 74219. Different letter indicates significant statistical differences (*p* < 0.05).

Then, the influence of the incubation time in a buffer solution (pH 7.0) before the extraction with ethanol was evaluated on untreated and PEF-treated cells of both *X. dendrorhous* strains. The results are shown in Figure 3. It can be observed that the incubation in the buffer solution was ineffective for extracting carotenoids in the PEF-treated cells of CECT 11028 strain and untreated cells of both strains. For such cases, extracted carotenoids never reached values over 0.25 ± 0.01 mg/g d.w. throughout all evaluated incubation time. Nevertheless, mutant PEF-treated cells resulted in a subsequent extraction yield of 0.88 ± 0.10 mg/g d.w. after 3 h of incubation, reaching a maximum value after 12 h of incubation (1.9 ± 0.18 mg/g d.w.). Extraction yields obtained at 24 h of incubation were not significantly different from those obtained at 12 h (*p* < 0.05). The extraction achieved after 12 h of incubation represented an improvement of more than twice the extraction yield achieved at the first 3 h of incubation and around 9.5-fold compared with untreated samples.

Since only the pre-treatment by PEF for carotenoids extraction was effective for *X. dendrorhous* ATCC 74219, only this strain was selected for subsequent studies in this section. Thereafter, the intensity of the PEF treatment and extraction time in ethanol effect on the carotenoids recovery yield was evaluated. The incubation time in buffer was kept at 12 h for such studies.

**FIGURE 4:**
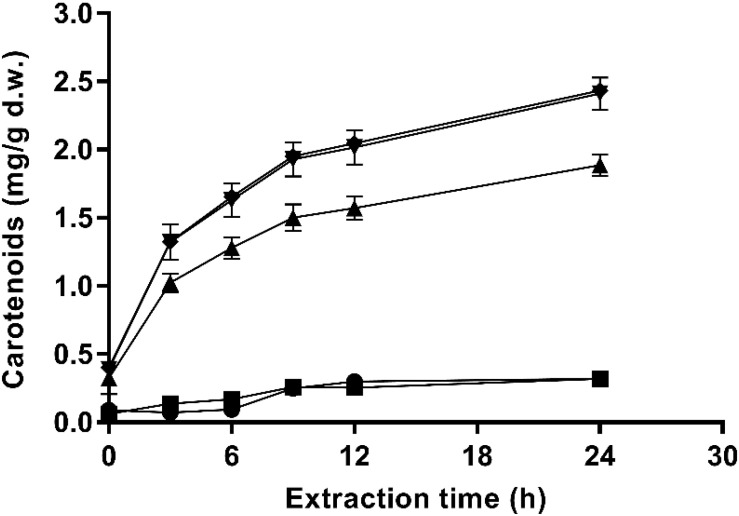
Effect of the electric field strength on ethanolic extraction kinetics of carotenoids from and *X. dendrorhous* ATCC 74219 after 12 h of incubation in buffer of pH 7.0: 10 kV/cm (■), 15 kV/cm (▲), 20 kV/cm (▼), 25 kV/cm (◆), and untreated (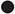).

The extraction kinetics in ethanol of carotenoids from untreated and PEF-treated cells at different pulsed electric field strength (135 μs) are shown in Figure 4. Untreated cells and PEF-treated with the lowest applied electric field (10 kV/cm) resulted in poor extraction yields. However, significant extraction levels were obtained at electric field strength equal to or higher than 15 kV/cm. These results confirmed that it was required the irreversible electroporation of the cell membranes to be able to improve carotenoids yields from fresh biomass of *X. dendrorhous* ATCC 74219 after buffer incubation. On the other hand, an increment in the electric field strength from 20 to 25 kV/cm did not significantly affect the extraction yield, supporting the fact that no statistically significant differences in the number of inactivated cells were observed at this electric field strength levels during 135 μs (Figure 1B).

Additionally, the extraction pattern depicted in Figure 4 shows a typical profile for solid-liquid extraction processes. The highest extraction rate was observed during the first 9 h of the process (around 80% of the total carotenoids were extracted). Then, the equilibrium seems to be reached at a lower rate.

The carotenoid extractability and trans-astaxanthin yields and the trans-astaxanthin percentage of recovered carotenoids obtained at each evaluated electric field strength levels after 12 h of buffer incubation and 24 h extraction with ethanol are shown in Table 1. Results for untreated samples (control), total carotenoids (extracted with DMSO) and the total specific energy applied at each electric field strength level are also shown. Total carotenoid yield recovered with the conventional method (DMSO) was 3.50 ± 0.15 mg/g d.w. Carotenoids yields ranged between 0.32 ± 0.02 and 2.43 ± 0.05 mg/g d.w. for PEF-treated cells, which corresponded to 9–70% of extractability. The carotenoid yield obtained with the control (untreated/ethanol) was 0.32 ± 0.02 mg/g d.w. (9% extractability). Thus, there was not a significant difference between the control and PEF-treated cells with an electric field level of 10 kV/cm. The highest carotenoid extraction yield was observed by applying an electric field strength in the range of 20–25 kV/cm and 135 μs, representing an extractability value of 70% of total carotenoids contained in the yeast suspension. However, at 20 kV/cm implies that 37% less energy is required in comparison with treatment at 25 kV/cm.

**TABLE 1:** Astaxanthin quantification by HPLC of ethanolic carotenoid extracts obtained from PEF-treated cells of *X. dendrorhous* ATCC 74219 at different treatment intensities and specific energy applied after 12 h of incubation in buffer of pH 7.0.

**Electric field strength (kV/cm)**	**Energy applied (KJ/kg)**	**Yield, carotenoids (mg/g)**	**Extractability (%)**	**Yield, *trans* astaxanthin (mg/g)**	***Trans-*astaxanthin (%)**
10.00	13.50	0.32 ± 0.02^a^	9 ± 0.54^a^	0.32 ± 0.05^a^	100 ± 0.0^a^
15.00	31.20	1.89 ± 0.08^b^	54 ± 2.24^b^	1.54 ± 0.17^b^	82 ± 0.84^b^
20.00	54.00	2.41 ± 0.12^c^	69 ± 3.37^c^	1.92 ± 0.01^c^	80 ± 0.38^c^
25.00	85.73	2.43 ± 0.05^c^	70 ± 1.29^c^	2.06 ± 0.02^c^	84 ± 0.38^c^
Control		0.32 ± 0.02^a^	9 ± 0.54^a^		
Total carotenoids (DMSO)		3.50 ± 0.15^d^	100 ± 0.0^d^	2.81 ± 0.18^d^	80 ± 1.69^d^

*Results are the average values of three independents experiments (mean ± standard deviation). ^*a*–*d*^Different letter indicates significant statistical differences (*p* < 0.05).*

HPLC analysis of the carotenoid extracts showed that from the recovered carotenoids, 80–84% corresponded to *trans-*astaxanthin for PEF-treated samples (Table 1). Similar% *trans-*astaxanthin was obtained for the total carotenoids extracted with DMSO (80%).

### Understanding Mechanism Involved in the Extraction Assisted by PEF of Carotenoid From *Xanthophyllomyces* Strains

After demonstrating the efficacy of ethanol for extracting large amounts of carotenoids from PEF-treated *X. dendrorhous* ATCC 74219 previously incubated in a buffer (pH = 7.0), studies were conducted in order to get an insight into the mechanisms involved and to be able to explain the inefficiency of PEF-treatment on the wild strain.

In order to verify if the PEF treatment triggered an enzymatic process that enabled the carotenoid extraction of *X. dendrorhous*, the β-glucanase and esterase activities were measured during the incubation of the untreated and PEF-treated cells (20 kV/cm and 135 μs) in a buffer of pH 7.

The β -glucanase activity was not detected in the supernatant of the suspension containing untreated or PEF-treated cells of both *X. dendrorhous* strains after 12 h of incubation at 25°C (data not showed).

**FIGURE 5:**
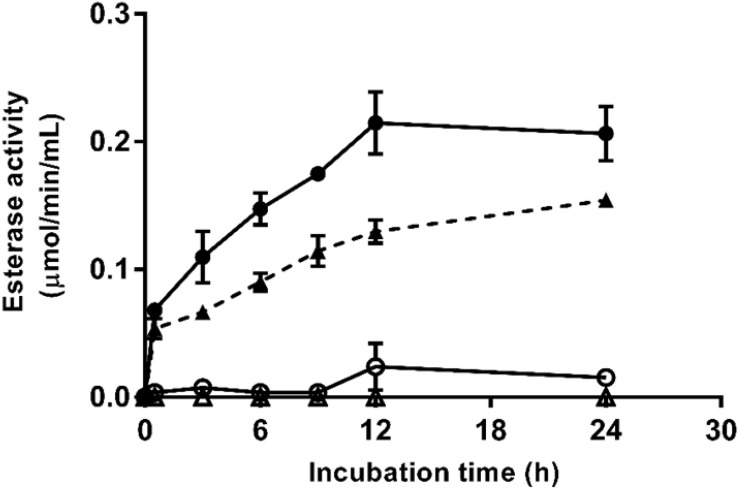
Esterase activity during aqueous incubation at 25°C of untreated (△) and PEF-treated (▲) *X. dendrorhous* CECT 11028 cells and untreated (○) and PEF-treated (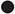) *X. dendrorhous* ATCC 74219 cells.

Enzymatic esterase activity during aqueous incubation at 25°C of untreated and PEF-treated *X. dendrorhous* ATCC 74219 and CECT 11028 cells are shown in Figure 5. Esterase activity in the media containing untreated cells was negligible even after 24 h of incubation. In contrast, esterase activity in the media containing PEF-treated cells of both *X. dendrorhous* strains increased along the time, reaching the maximum activity and equilibrium after 12 h of aqueous incubation, approximately. Such results suggested that the enzyme esterase was released from the cytoplasm of the treated cells as consequence of the electroporation caused by PEF. However, despite the higher enzymatic activity detected in the ATCC 74219 strain, the fact that esterase activity was detected during the aqueous incubation of both strains makes unclear whether the esterase enzyme is involved in the mechanisms of the extraction assisted by PEF.

**FIGURE 6:**
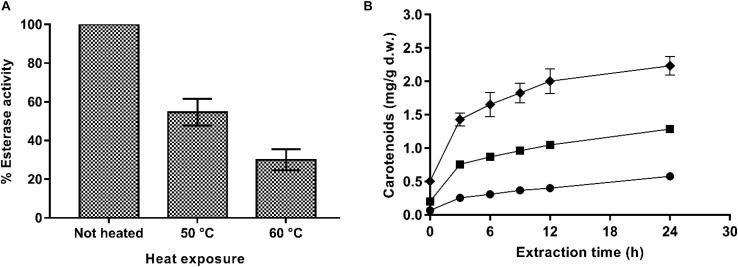
**(A)** Percentage of esterase activity remaining in the supernatant containing PEF-treated (20 kV/cm, 135 μs) *X. dendrorhous* ATCC 74219 cells after 5 min of heating at 50 and 60°C. **(B)** Effect of the thermal inactivation of esterase on ethanolic extraction of carotenoids from and *X. dendrorhous* ATCC 74219 after 12 h of incubation in buffer of pH 7.0: unheated (◆) and heat-treated at 50°C (■), 60°C (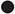). The 100% of esterase activity represent the maximum esterase activity quantified in the unheated PEF-treated cells supernatant.

Thus, to confirm this hypothesis, an experiment consisting of exposing the PEF-treated *X. dendrorhous* ATCC 74219 cells to a heat treatment before incubation in buffer of pH 7.0 to denaturalize esterase enzyme was conducted. Percentages of esterase activity remaining in the supernatant containing PEF-treated cells treated for 5 min at 50 and 60°C after 12 h of incubation in buffer are shown in Figure 6A. Extraction curves of carotenoids from PEF-treated cells treated at 50 and 60°C after 12 h of buffer incubation and subsequent extraction in ethanol are compared with the carotenoid extraction from unheated cells in Figure 6B.

Results showed that the thermal treatment effectively inactivated the esterase enzyme, decreasing its activity in the supernatant by 50 and 75% for the treatments applied at 50 and 60°C, respectively. Accordingly, carotenoid recovery was much lower for the heat-treated PEF-treated cells along all the extraction process. After 24 h of extraction, a decrease by 42 and 72% in the extraction yield was observed for thermally treated cells at 50 or 60°C, respectively.

The correlation between carotenoid extraction yield and the esterase activity reduction in the supernatant containing PEF-thermally treated cells would confirm that PEF assisted extraction of carotenoids was a process mediated by esterases at the evaluated conditions at least for from *X. dendrorhous* ATCC 74219. However, these results are in contrast to those observed in *X. dendrorhous* CECT 11028, where the esterase activity detected in the PEF-treated cells were not correlated with carotenoids extraction.

**FIGURE 7:**
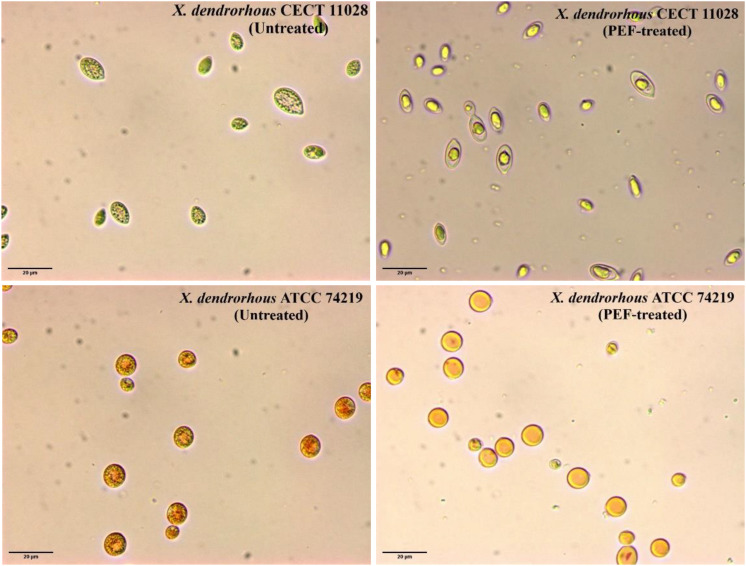
Optical microscopy observation (600x) of untreated and PEF-treated of *X. dendrorhous* CECT 11028 and *X. dendrorhous* ATCC 74219 cells after 12 h of incubation at 25°C in a buffer of pH 7.

An optical microscopy comparation after 12 h of incubation at 25°C in a buffer of pH 7.0 of untreated and PEF-treated cells of the two strains of *X. dendrorhous* is shown in Figure 7. It was observed at the beginning of the incubation (data not shown), that carotenoids were located in the cytoplasm into droplet structures. Incubation for 12 h did not cause any significant change in the untreated cells of both strains. Furthermore, optical observation confirmed the higher carotenoid production by *X. dendrorhous* ATCC 74219 (3.5 ± 0.15 mg/g d.w.) compared to *X. dendrorhous* CECT 11028 (0.36 ± 0.02 mg/g d.w.). While in the hyper-producer most of the observed droplets corresponded to carotenoids in the other strain most droplets correspond to lipids.

On the other hand, significant differences were observed between the two strains when the PEF-treated cells were incubated in pH 7.0 buffer. While complete droplet disintegration was observed in *X. dendrorhous* ATCC 74219, in the case of the strain CECT 11028 incubation of the PEF-treated cells clearly led to lipid droplet fusion in a bigger droplet. In both cases, the changes in the droplets observed after incubation could be mediated by esterases, which activity was triggered by the electroporation of the cell membrane of the yeast. These observations seem to indicate that ethanol was only effective for extracting those intracellular carotenoids that are free in the cytoplasm. However, ethanolic extraction was ineffective when the carotenoids were located into droplet structures such as in untreated or PEF-treated cells before incubation in both strains or as in the CECT 11028 strain after 12 h of incubation.

## Discussion

Pulsed electric fields-assisted extraction of intracellular compounds is associated with the improvement of mass transfer processes trough cytoplasmic membrane once its permeability has been modified by electroporation (Puértolas et al., 2012). It is well known that irreversible modification of the selective permeability of the cytoplasmic membrane leads to the microbial inactivation (Pillet et al., 2016). Therefore, in order to identify the appropriate range of treatment conditions for irreversible electroporation, the sensitivity of the two strains to PEF treatments of different intensity was investigated (Figure 1). *X. dendrorhous* ATCC 74219 strain was more PEF resistant than the strain CECT 11028 and compared with other yeast, both strains showed lower sensitivity to PEF (Aronsson et al., 2005; Martínez et al., 2016). Currently, the influence of the cell envelopes surrounding the cytoplasmatic membrane on electroporation by PEF is unclear, but it has suggested that cell wall could influence the sensitivity to PEF (Aronsson et al., 2005; García et al., 2007). The highest PEF resistance could be related with the fact that oleaginous yeasts such as of *X. dendrorhous* have a higher fraction of chitin and/or mannan in the cell wall compared to non-oleaginous species that contribute to the rigidity and resistance to cell lysis (Khot et al., 2020). This rigidity and resistance to cell lysis should be higher in the ATCC 74219 strain to contain around 10 times more of carotenoids than those contained in the CECT 11028.

A small proportion of inactivated *X. dendrorhous* cells were impermeable to PI after application of mild intensity PEF treatments that caused low inactivation. This observation, previously reported by other authors, suggests that in these cells, the pore resealing is a physical process rather than a process with biosynthetic requirements (Martínez et al., 2018). Therefore, quantification of the proportion of inactivated cells is a suitable index for estimation of the number of irreversible electroporation cells when the inactivation is higher of a given value, that in the case of the strains of *X. dendrorhous* investigated was around 70%.

In order to develop a sustainable and cost-efficient overall bioprocess, carotenoids were extracted from wet biomass of *X. dendrorhous* (avoiding too high costs derived from drying) and ethanol was used as a solvent. Ethanol is a polar water-soluble solvent that is less toxic and greener than others used for extracting lipophilic compounds such as hexane, dichloromethane or chloroform (Saini and Keum, 2018).

The water solubility of ethanol in water should allow its diffusion through the cell wall and cell membrane and reaching the carotenoids located in the cytoplasm of *X. dendrorhous.* However, ethanol was inefficient for extracting carotenoids from the two investigated strains, even when around 80% of the population cells were irreversibly electroporated. This observation suggested that ethanol was not able to dissolve the carotenoids located in the droplets.

As it was previously mentioned, it was suggested that PEF triggers an enzymatic process that enables the yeast autolysis, which then allows the extraction of the intracellular compounds. Thus, the subsequent studies aimed at verifying this hypothesis and increasing the recovery yields of carotenoids. By evaluating the incubation process in the buffer solution, it was shown that ethanol was effective for extracting carotenoids from PEF-treated cells of *X. dendrorhous* ATCC 74219. Recently, several authors have reported that an aqueous incubation period after PEF treatment increased the extraction yield of carotenoids, lipids and proteins from microalgae and yeasts (Luengo et al., 2014; Dimopoulos et al., 2018; Martínez et al., 2018, 2019; Silve et al., 2018; Scherer et al., 2019). This improvement in the extraction has been attributed to enzyme-driven processes triggered by PEF that occur during the aqueous incubation period.

The efficiency of ethanol for extracting carotenoids from PEF-treated *X. dendrorhous* ATCC 74219 after buffer incubation was dependent on the proportion of cells electroporated. Increments in the electric field strength that lead to a high proportion of electroporated cells result in a higher extraction until 20 kV/cm.

HPLC quantification of astaxanthin from ethanolic extracts obtained with different PEF treatment intensities (Table 1) showed that up to 84% (2.06 ± 0.02 mg/g d.w.) of total carotenoids extracted corresponded to *trans-*astaxanthin. This proportion agrees with values reported in the literature for other *X. dendrorhous* strains (Johnson and Lewis, 1979; de la Fuente et al., 2010; Schmidt et al., 2011). The high yield of *trans-*astaxanthin (2.06 ± 0.02 mg/g d.w.) obtained in this study from *X. dendrorhous* ATCC 74219 was in range to other reported yields obtained for *X. dendrorhous* ATCC 74219. Amado and Vázquez (2015) reported a maximum astaxanthin specific concentration of 2.0 mg/g d.w. by culturing the same strains of *X. dendrorhous* ATCC 74219 in a low-cost media designed from marine by-products, while Stoklosa et al. (2018) reported 2.49 mg/g d.w. specific astaxanthin production by culturing the same strain in sweet sorghum juice.

The results related with the evaluation of enzymatic activities seemed to confirm that the enzymatic esterase activity triggered by PEF mediates the effective ethanolic extraction of carotenoids from PEF-treated *X. dendrorhous* ATCC 74219 at the evaluated conditions. The uncontrolled molecular transport thought the electroporated cytoplasmic membrane of the yeast could decrease the osmotic pressure of the cytoplasm as a consequence of water inlet, causing the plasmolysis of lysosomes and the liberation of hydrolytic enzymes such as esterases. These esterases could hydrolyze the triacylglycerols of lipid droplets resulting in the loss of their structure and the subsequent carotenoid release. Once the carotenoids are free in the cytoplasm, they could be dissolved in ethanol and the complex carotenoid-solvent could diffuse across the cell membrane driven by a concentration gradient. The presence of esterase activity in the supernatant containing PEF-treated cells during buffer incubation and the lower carotenoid extraction yield when esterase activity was reduced by heating, supported such hypothesis. Furthermore, the lack of effectivity in the carotenoid extraction when PEF-treated cells were immediately incubated in ethanol could be explained for esterase denaturation when exposed to high concentrations of ethanol.

Although esterase activity was also detected in the electroporated *X. dendrorhous* CECT 11028, the inefficiency of ethanol for extracting the carotenoids could be related to the changes observed in the cytoplasm of the cells after incubation (Figure 7). As it has been previously described for the microalga *Chlamydomonas reinhardtii*, the aqueous incubation of the PEF-treated cells clearly led to the fusion of the lipid droplets in a big globule (Bodénès et al., 2016; Bensalem et al., 2018). In addition, this big globule of lipids might content the synthesized carotenoids by the strain CECT 11028 because neither free carotenoids nor carotenoid droplets were observed in the cytoplasm after fusion. Lipid fusion of the lipid droplets did not occur in the strain ATCC 74219 probably because, as it is observed in Figure 7 in this hyper-producer strain, most of the droplets contained in the cytoplasm correspond to carotenoids.

Due to polarity properties of ethanol, this solvent was unable to dissolve the single large lipid droplet, mainly constituted for neutral lipids with long hydrophobic fatty acid chains (Halim et al., 2012). Therefore, using a mixture of polar and non-polar solvents might allow dissolving this lipid structure, or the rupture of the cell wall should be required for carotenoid extraction from the CECT 11028 strain (Bensalem et al., 2018).

## Conclusion

This research has demonstrated that application of PEF treatment and subsequent aqueous incubation of fresh biomass of *X. dendrorhous* ATCC 74219 allowed the ethanolic extraction of 70% of the total carotenoids which up to 84% correspond to *trans-*astaxanthin. The effective extraction depended on the proportion of electroporated cells and the hydrolytic activity of the enzyme esterase during incubation triggered by PEF. The ineffective extraction of carotenoids from *X. dendrorhous* CECT 11028 seemed to be related to the inability of ethanol to dissolve the lipids structure containing carotenoids.

In order to establish the procedure developed in this study as a sustainable method with low environmental impact for extraction of *trans-*astaxanthin, further studies are required to optimize PEF treatment, enzymatic activity and extraction conditions for improving extraction yields with the lowest energetic costs.

## Data Availability Statement

The raw data supporting the conclusions of this article will be made available by the authors, without undue reservation.

## Author Contributions

DA-M, JM, and JR: conception and design of the study. DA-M, CD, and JMM: acquisition of data. DA-M, LM-O, and JR: analysis and interpretation of the data. DA-M and JMM: drafting of the manuscript. LM-O, JM, and JR: critical revision of the manuscript. All authors contributed to the article and approved the submitted version.

## Conflict of Interest

The authors declare that the research was conducted in the absence of any commercial or financial relationships that could be construed as a potential conflict of interest.
